# Brain-targeted delivery of Valsartan using solid lipid nanoparticles labeled with Rhodamine B; a promising technique for mitigating the negative effects of stroke

**DOI:** 10.1080/10717544.2023.2179127

**Published:** 2023-02-15

**Authors:** Shereen A. Sabry, Amal M. Abd El Razek, Mohamed Nabil, Shaimaa M. Khedr, Hanan M. El-Nahas, Noura G. Eissa

**Affiliations:** a Department of Pharmaceutics, Faculty of Pharmacy, Zagazig University, Zagazig, Egypt; b Pharmacology Department, Faculty of Pharmacy, New Valley University, Kharga, Egypt; c Pharmaceutical and Fermentation Industries Development Centre (PFIDC), City of Scientific Research and Technological Applications (SRTA-City), New Borg El-Arab, Alexandria, Egypt; d Science Academy, Badr University in Cairo, Badr City, Cairo, Egypt

**Keywords:** Valsartan, solid lipid nanoparticles, stroke, factorial design, transmission electron microscopy, photon imaging

## Abstract

The brain is a vital organ that is protected from the general circulation and is distinguished by the presence of a relatively impermeable blood brain barrier (BBB). Blood brain barrier prevents the entry of foreign molecules. The current research aims to transport valsartan (Val) across BBB utilizing solid lipid nanoparticles (SLNs) approach to mitigate the adverse effects of stroke. Using a 3^2^-factorial design, we could investigate and optimize the effect of several variables in order to improve brain permeability of valsartan in a target-specific and sustained-release manner, which led to alleviation of ischemia-induced brain damage. The impact of each of the following independent variables was investigated: lipid concentration (% w/v), surfactant concentration (% w/v), and homogenization speed (RPM) on particle size, zeta potential (ZP), entrapment efficiency (EE) %, and cumulative drug release percentage (CDR) %. TEM images revealed a spherical form of the optimized nanoparticles, with particle size (215.76 ± 7.63 nm), PDI (0.311 ± 0.02), ZP (-15.26 ± 0.58 mV), EE (59.45 ± 0.88%), and CDR (87.59 ± 1.67%) for 72 hours. SLNs formulations showed sustained drug release, which could effectively reduce the dose frequency and improve patient compliance. DSC and X-ray emphasize that Val was encapsulated in the amorphous form. The *in-vivo* results revealed that the optimized formula successfully delivered Val to the brain through intranasal rout as compared to a pure Val solution and evidenced by the photon imaging and florescence intensity quantification. In a conclusion, the optimized SLN formula (F9) could be a promising therapy for delivering Val to brain, alleviating the negative consequences associated with stroke.

## 1. Introduction

Stroke is the most common cause of permanent disability in adults worldwide and the second greatest cause of death in industrialized countries (Tapeinos et al., 2017). It has been found that stroke is accompanied by an increased level of angiotensin II and angiotensin II type-1 (AT1) receptor, and the outcome of stroke is determined by the volume of the ischemic core, the extent of secondary brain damage which manifested by brain swelling, and impaired microcirculation and inflammation (Barakat et al., 2014).

Angiotensin receptor blockers (ARBs) have been shown to ameliorate peripheral and central actions of angiotensin II, mediated by AT1-receptors, and also to stimulate unopposed angiotensin II type 2 (AT2) receptors that are up-regulated in ischemic area (Barakat et al., 2014; Pai et al., 2016). Additionally, it has been confirmed in large clinical trials, that ARBs demonstrate an essential role in preventing both primary and secondary stroke (Dahlof, 2009).

The blood-brain barrier (BBB) is a highly effective system that separates the central nervous system (CNS) from general circulation. The capacity of a certain molecule to cross BBB is a critical prerequisite in the formulation of any drug targeting the CNS. Basically, it promotes selective transport of essential molecules for brain function (Morsi et al., 2013).

Therefore, screenings that predict BBB permeability of candidate compounds are indispensable for enhancing the field of drug discovery and finding effective therapeutics for many CNS related diseases. Although, there is a lot of ongoing research to evaluate BBB permeability, yet it is time-intensive and inefficient (Tang et al., 2022).

Valsartan (Val) is one of ARBs that is available in both solution and tablet dosage forms, however, it is a tetrazole derivative that is slightly soluble in water with bioavailability of about 25% and a volume of distribution of 17 liters. This indicates that Val does not distribute extensively into tissues (Michel et al., 2013), and as a result, it either crosses the BBB to a very low extent or not at all (Michel et al., 2016). Hence, there was a strong demand for enhancing Val delivery to brain.

One possible approach for circumventing the BBB is through the use of SLNs (He et al., 2019). It is among the safest and most cost-effective drug carriers, allowing for the nontoxic and successful treatment of neurological illnesses. SLNs are simply made up of a drug encapsulated within a lipid core and a surfactant in the outer coat. Accordingly, SLNs are considered an excellent alternative to polymeric systems with minimal possible toxicity (Ghorab et al., 2015).

The intranasal route allows drugs to be delivered directly to the CNS. So, drugs loaded into SLNs can directly penetrate the BBB from the nasal cavity (Duong et al., 2020).

This work attempted to improve Val aqueous solubility and enhance its transport across the BBB by loading it into SLNs designed to be delivered through the nasal cavity. The efficiency of the drug being delivered to the brain was explored by tracing fluorescently labeled nasal Val-SLN with rhodamine b. (Rh-B). Namely, drug deposition in the brain was detected by imaging and quantifying fluorescence intensity (Photon Imager optima - Bio space lab, France, Software version: 3.5.10.1464).

The procedure was modeled using a three-factor two-level (2^3^) full factorial design (8 runs), design was employed to investigate the impact of three independent variables, namely lipid concentration (X1), surfactant concentration (X2), and homogenization speed (X3), on four dependent variables, namely particle size (PS-Y1), zeta potential (ZP-Y2), entrapment efficiency % (EE-Y3), and cumulative drug release % (CDR).

## 2. Materials and methods

### 2.1. Materials

Valsartan (Val) was obtained as a gift sample from Servier Egypt Industries, Egypt. Glyceryl monostearate (GMS), Poloxamer 407 (P407), and egg lecithin were kindly provided by Egyptian International Pharmaceutical Industries Co., (EIPICO.), Egypt. Rhodamine B (Rh-B) was purchased from Lab vision Trade Company, Egypt. Chloroform, acetone, disodium hydrogen phosphate, and potassium dihydrogen phosphate were purchased from El-Gomhouria Company for trading chemicals and medical appliances, Cairo, Egypt.

### 2.2. Methods

#### 2.2.1. Preparation of Val-loaded SLNs

Valsartan loaded SLNs were prepared by emulsification solvent evaporation process (Palei & Das, 2013) with slight modifications (volume and type of organic solvent and stirring rate). Briefly, Val (1% w/v) was dissolved in a mixture of chloroform: acetone (5 ml: 2 ml) in which GMS and egg lecithin were previously dissolved by stirring at 40 °C using a magnetic stirrer. This oily phase was then dripped into 25 ml of an aqueous P407 solution kept at the same temperature (40 °C) and emulsified by homogenization for 15 minutes to prepare O/W emulsion. The formed dispersion was stirred continuously at 700 rpm for 3.5 h using a mechanical stirrer to assure complete evaporation of the organic solvent residue. The lipid was precipitated out in the aqueous medium, resulting in SLN formation. Thereafter, the formulations were sonicated for 4 minutes by a pan sonicator and kept at room temperature overnight. Then, they were stored in the refrigerator for further studies.

Plain SLNs were prepared by the same procedure mentioned above, but without the addition of the drug. These plain SLNs were used as a blank.

To prepare Val-SLNs coupled with Rh-B for *in-vivo* fating, Rh-B (1 mg/100 mg lipid) was added at the lipid phase step, and the procedure was completed in the same way as previously mentioned (Topal et al., 2020). Eight formulations of SLNs (F1 to F8) were prepared according to 2^3^ full-factorial experimental design as represented in Table 1.

**Table 1.** Formulation parameters.

Formulation code	Lipid % (w/v)	SAA % (w/v)	Homogenization speed (rpm)
F1	3	0.5	10,000
F2	3	0.5	15,000
F3	3	1.5	10,000
F4	3	1.5	15,000
F5	5	0.5	10,000
F6	5	0.5	15,000
F7	5	1.5	10,000
F8	5	1.5	15,000

#### 2.2.2. Characterization of Val-loaded SLNs

##### 2.2.2.1. Particle size (PS), polydispersity index (PDI) and zeta potential (ZP)

The particle size, PDI, and zeta potential of different SLN formulations were measured by dynamic light scattering (DLS) technique (Malvern Zetasizer Nano–ZS90). A fixed volume of each SLN formulation was diluted with distilled water and then injected into a clear disposable zeta cell (Kaur et al., 2016a; Ibrahim et al., 2019). Results were presented as mean values ± standard deviation (SD).

##### 2.2.2.2. Entrapment efficiency (EE) %

A dialysis technique was employed to separate the free drug from Val-loaded SLNs. A certain volume of SLNs dispersion equivalent to (40 mg) Val was placed into a dialysis bag (molecular weight cutoff 12,000–14,000 Da) previously soaked in Sörensen phosphate buffer solution (PBS) of pH 6.4 overnight and then immersed in a screw-capped bottle containing 100 ml of PBS (pH 6.4). The entire system was kept at 25 °C with continuous stirring in a thermostatic shaker water bath (Kotterman Shaker D3165 Hangisen, W-Germany) at 100 rpm.

The free drug was dialyzed for one hour each time against 100 ml of PBS (pH 6.4) and assayed at *λ* max of 242 nm for Val content. The procedure was repeated till there was no Val in the medium, and the total free Val is the sum of all readings (Tamizharasi et al., 2009).

The percentage of EE of SLN formulations was determined using the following equation (Alajami et al., 2022).

$$\mathrm{EE}\;(\%)\;=\frac{\text{Total amount of Val added}-\text{ Amount of free Val}}{\text{Total amount of Val }}\times 100$$

##### 2.2.2.3. In-vitro cumulative drug release study (CDR%)

*In-vitro* Val release study was conducted for pure Val solution in PBS (pH 6.4) and all formulations of SLNs for 72 hours, using the dialysis bag technique (Misra et al., 2016; Chandana et al., 2021). Dialysis bags were soaked in PBS (pH 6.4) overnight before use. Fixed volume of pure drug solution and SLN formulations equivalent to (40 mg Val) were transferred to dialysis bags, the two ends firmly sealed and then suspended in a preheated receptor medium (100 ml PBS of pH 6.4) at 37 ± 0.5 °C under stirring at 100 rpm in a thermostatic shaking water bath. An aliquot of the dissolution medium (3 ml) was withdrawn at different time intervals, passed through a 0.22 µm filter, and replaced with an equal volume of fresh medium to maintain a constant volume. The drug concentration in each aliquot was analyzed by UV spectroscopy at 242 nm. All measurements were performed in triplicate, and the cumulative drug release percent (CDR%) was represented as mean ± SD.

##### 2.2.2.4. Kinetic study of drug release

The release data of all SLN formulations were subjected to explore the mechanism of release kinetics according to the following models: zero order model (Q_t_ = Ko._t_), first order model (log Q_t_ = log Q_o_ – K._t_/2.303), Higuchi release model (Q_t_ = K_H_.t^0.5^), Korsmeyer-Peppas model (Q_t_/Q_∞_ = K_k_.t^n^) and Hixson–Crowell model (Q_o_^1/3^ – Q_t_^1/3^ = K_s_.t); where, Q_t_: amount of drug released, t: time interval, Q_o_: initial drug amount, Q_∞_: the amount of drug released at time infinity (∞), K_o_, K, k_H_, K_s_, K_k_: release rate constants and n: release exponent (El-Nahas, 2010; Sheshala et al., 2019).

The highest correlation coefficients (R^2^) referred to the drug release order, which was further confirmed by the release exponent value (n) of the Korsmeyer-Peppas model (Ibrahim et al., 2021).

##### 2.2.2.5. Stability study of SLN formulations

All prepared SLNs formulations were stored at refrigerator temperature (4 °C) for 4 months. At the end of this period, the means of PS, PDI, and ZP were measured (Palei & Das, 2013).

##### 2.2.2.6. Statistical analysis

Student’s *t*-test and one-way ANOVA were adopted to assess the significance of the difference between different formulations using Graph-Pad Prism version 5.02. Values were represented as the mean ± SD (Hasan et al., 2020; Hassan et al., 2020; Nair et al., 2021).

##### 2.2.2.7. Optimization of Val-loaded SLNs

Formula optimization was conducted by factorial design software. The optimization strategy was reliant on the preferred target of each response (P.S = 150 nm, ZP = −20 mV, EE% = 60, and CDR% = 90). The values suggested by the software to prepare the optimized formulation (F9) were 4.9379% w/v lipid, 0.6507% w/v SAA, and 10000 rpm as homogenization speed.

##### 2.2.2.8. Differential scanning calorimetry (DSC)

The melting and crystallization behavior of pure Val, P407, egg lecithin, GMS, and optimized Val-loaded SLN (F9) were studied by DSC (DSC-60; Shimadzu Corporation, Tokyo, Japan). For each measurement, accurately weighed samples (2 mg) were sealed in aluminum pans and analyzed over a temperature range of 0-250 °C under a nitrogen purge (50 ml/min) with a heating rate of 10 °C/min.

##### 2.2.2.9. Fourier transform infrared (FTIR) spectroscopy

FTIR analysis was performed for the same components as in DSC analysis. An FTIR spectrometer (Perkinelmer 1600 FTIR spectrophotometer, USA) was used to record the FTIR spectra between 4.000 and 500 cm^−1^ using the KBr.

##### 2.2.2.10. X-ray diffraction (XRD) study

The encapsulation of the drug inside the nanoparticles was further confirmed by XRD (Ultima IV; Rigaku Corporation, Tokyo, Japan, using a Goniometer PW18120 as a detector). Samples were exposed to Cu·Kα radiation (40 kV, 25 mA, k = 0.15418 nm) and analyzed at (2θ) from 10° to 80°. Bragg’s equation was used to transform the data from scattering angle to the spacing of lipid chains.

##### 2.2.2.11. Transmission electron microscopy (TEM)

The morphology of F9 with and without Rh-B coupling was investigated using TEM (Model JEM-1230, JOEL, Tokyo, Japan), in which a few drops of the formula were mounted on a carbon-coated grid, left for 2 minutes to allow better adsorption on the carbon film, excess liquid was removed with a filter paper, and then a drop of phospho-tungstic acid (1%) was added (Kurakula et al., 2016; Al Ashmawy et al., 2021).

#### 2.2.3. In-vivo study of optimized SLN formulation

Male albino mice (weighing 25 ± 3 g) and aged 12 weeks were obtained from VACSERA (Giza, Egypt). Nude mice were chosen to allow the detection of faint light signals (Mannucci et al., 2020). Mice were accommodated for one week prior to the start of the experiments, which were conducted totally under the supervision of veterinary microsurgery and with the agreement of Zagazig University’s Animal Ethics Committee (ZU-IACUC) under approval protocol No, ZU-IACUC/3/F/106/2020.

##### 2.2.3.1. Administration of studied formulations to animals

For ethical reasons, a small but statistically significant number of mice were used (Mannucci et al., 2020). Twenty mice were divided into four groups (5 mice per group). Group 1 received a vehicle (control group). Group 2 received the optimized Val-loaded SLN (F9), group 3 received blank SLN (F10), and group 4 received a pure Val solution (F11). All *in-vivo* tested formulations (F9, F10, and F11) were pigmented by Rh-B, as previously mentioned to be able to be tracked in the brain under *in-vivo* optical imaging (Aboud et al., 2016). Twenty microliters of each formulation were administered intranasally to mice, which was equivalent to 10 mg of valsartan per kg of mice (Sironi et al., 2004; Hadi et al., 2015).

The permanent stroke of the distal middle cerebral artery was induced using an electrothermic coagulator, as previously described by Llovera et al. (2014).

For surgical proceedings, the mice were anesthetized by i.p. administration of ketamine/xylazine cocktail at a dose level (0.1 ml and 0.1 ml/100 g body weight, respectively). Mice were shaved gently on the back area of the skull and an antisepsis of the area was performed with 4% alcohol-based iodine. At the place of the operation, a small incision was induced, then gently removing a piece of the skull to expose the middle cerebral artery and allow for the electro cautery drill. Post-surgically, all the animals were kept separately in their cages, and the wounds were cleaned daily without any dressing or covering over the wound. All formulations were administered by micropipette into both nostrils, following the protocol discussed by Hanson and coworkers, three days before stroke induction and continuing for another three days after stroke. At the end of the third day after stroke, mice were sacrificed ethically through an isoflurane overdose according to IACUC (Institutional Animal Care and Use Committee) recommendations. After perfusion, both lung and brain were excised and imaged (Hanson et al., 2013; Mannucci et al., 2020).

##### 2.2.3.2. Principles of photon imaging experiment

The noninvasive detection and quantification of fluorescence distributed throughout the isolated organs (brain and lung) were performed by (photon Imager optima - Bio Space Lab, France, Software Version: 3.5.10.1464), allowing the evaluation of the biodistribution of fluorescently labeled formulations (Mannucci et al., 2020). Fluorescent images were obtained for dissected brain and lung isolated from all groups at λex = 539 nm and λem= 615 nm.

## 3. Results and discussion

### 3.1. Particle size, polydispersity index and zeta potential

Results of PS, PDI and ZP are showed in Table 2.

**Table 2.** Particle size, PDI and zeta potential of SLNs formulations.

Formulation code	Particle size (nm)	PDI	Zeta potential (mv)
**F1**	99.05 ± 4.62	0.358 ± 0.03	−19.16 ± 0.37
**F2**	138.33 ± 1.89	0.528 ± 0.02	−18.66 ± 0.23
**F3**	1925.60 ± 75.07	0.716 ± 0.07	−17.56 ± 0.28
**F4**	639.46 ± 39.71	1 ± 0.00	−15.36 ± 0.51
**F5**	98.28 ± 5.63	0.259 ± 0.08	−17.66 ± 0.20
**F6**	101.89 ± 2.84	0.195 ± 0.03	−17.4 ± 0.36
**F7**	394.10 ± 18.37	0.191 ± 0.03	−16.76 ± 0.80
**F8**	213.63 ± 0.50	0.556 ± 0.03	−22.06 ± 0.66

#### 3.1.1. Particle size

The particle size (Y1) of all formulations was in the range of 98.28 nm to 1925.60 nm for F5 and F3, respectively. The influence of independent variables and their interactions on particle size could be identified by the following polynomial regression equation:

Y1 = 451 − 249 X1 + 342 X2−178 X3−240 X1*X2 + 134 X1*X3 − 189 X2*X3

**Figure 1.**
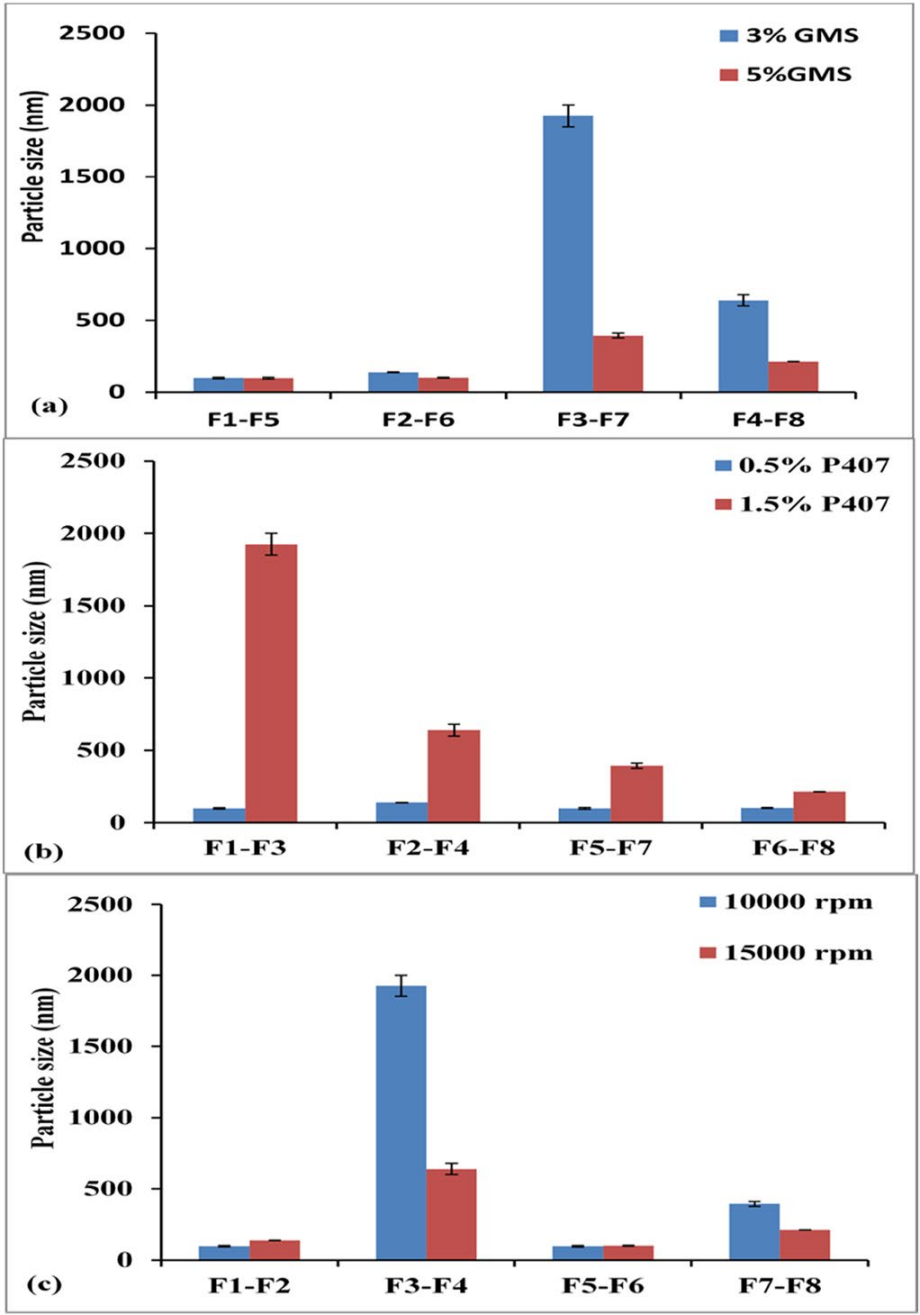
Effect of lipid % (a), SAA % (b) and homogenization speed (c) on particle size.

From the obtained results, it was observed that, when increasing the lipid concentration from 3 to 5 (% w/v), there was a strong significant negative correlation with particle size (Pearson coefficient (r) = −0.426 and P value = 0.014) (Gardouh et al., 2010). The particle size decreased from 138.33 ± 1.89 nm to 101.89 ± 2.84 nm (F2, F6), 1925.60 ± 75.075 nm to 394.10 ± 18.37 nm (F3, F7), and 639.46 ± 39.71 nm to 213.63 ± 0.503 nm (F4, F8) as elucidated in Figure 1a and Table 2. These results were in harmony with Steiner and Bunjes, who related this behavior to the non-linear increase in the viscosity of the continuous phase, which is inversely proportional to the droplet size (Steiner & Bunjes, 2021).

Another explanation might be that the size of SLN is affected by the number of carbon atoms in the fatty acid chain of the lipid. GMS had a smaller number of carbon atoms on its fatty acid chain, resulting in a smaller SLN. Therefore, increasing Glyceryl mono-stearate concentration resulted in the formation of small sized SLN (Gamal et al., 2020).

Increasing SAA concentration from 0.5 to 1.5 (% w/v) showed a strong significant positive correlation on particle size (r = 0.584 and P value < 0.0001). These results were in accordance with Soma et al. (2017). By the increase of SAA concentration from 0.5 to 1.5 (% w/v), a significant increase in particle size was noticed from 99.05 ± 4.62 nm to 1925.60 ± 75.075 nm (F1, F3), 138.33 ± 1.89 nm to 639.46 ± 39.71 nm (F2, F4), 98.28 ± 5.63 nm to 394.10 ± 18.37 nm (F5, F7) and 101.89 ± 2.84 nm to 213.63 ± 0.503 nm (F6, F8) as obvious in Figure 1b. These results were matched with Alajami and coauthors, who attributed these findings to the accumulation of excess SAA molecules at the nanoparticle surface or due to the expansion of the interfacial film by increasing SAA concentration (Asasutjarit et al., 2007; Alajami et al., 2022). Another justification for increasing particle size by increasing SAA concentration is the dehydration of propylene oxide and ethylene oxide blocks within the poloxamer molecule during emulsification and hot homogenization, leading to a reduction of steric repulsion (Gamal et al., 2020).

Results in Figure 1c verified that, when homogenization speed was increased from 10,000 to 15,000 rpm at constant lipid and SAA concentration, particle size decreased significantly from 1925.60 ± 75.07 nm to 639.46 ± 39.71 nm (F3, F4) and 394.10 ± 18.37 nm to 213.63 ± 0.503 nm (F7, F8). This result might be due to inefficient speed to reduce the particles at a lower speed. Whereas, the high-speed homogenization was sufficient to decrease the particle size through the high intensity of the shearing force acting on the particles (Kushwaha et al., 2013).

#### 3.1.2. Polydispersity index

**Figure 2.**
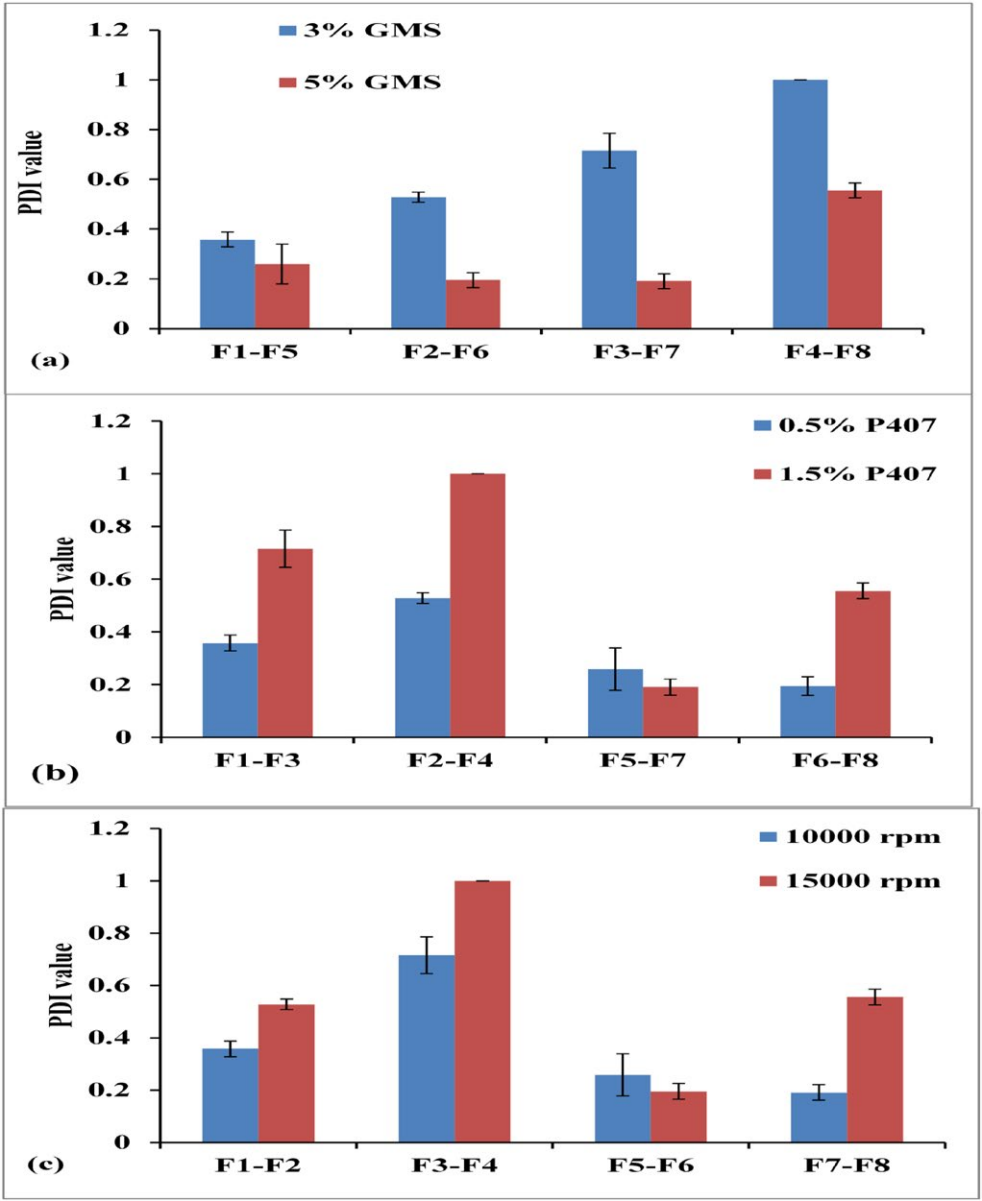
Effect of lipid % (a), SAA % (b) and homogenization speed (c) on PDI values.

Polydispersity index is a measurement of the broadness of the particle size distribution. Values which less than 0.5 are usually accepted by researchers, while 0.3 and below are most favorable (Hassan et al., 2020). PDI values of all SLNs ranged between 0.191 ± 0.036 and 1 ± 0.00 for F7 and F4, respectively as shown in Figure 2. F4, F5, and F6 had PDI< 0.3, indicating a homogeneous population of lipid vesicles (Danaei et al., 2018).

Figure 2a clarified that, increasing the lipid concentration from 3 to 5%, led to a significant decrease in PDI from 0.358 ± 0.03 to 0.259 ± 0.08 (F1, F5), 0.528 ± 0.02 to 0.195 ± 0.03 (F2, F6), 0.716 ± 0.07 to 0.191 ± 0.03 (F3, F7), and from 1 ± 0.00 to 0.556 ± 0.03 (F4, F8). This could be due to the reduction in particle size upon increasing lipid content. This was in accordance with Suhaimi and coworkers, who found that a decrease in the particle size was associated with a reduction in PDI values (Suhaimi et al., 2015).

The increase in SAA concentration from 0.5 to 1.5%; resulted in an increase in PDI from 0.358 ± 0.03 to 0.716 ± 0.07 (F1, F3), 0.528 ± 0.02 to 1 ± 0.00 (F2, F4), and 0.195 ± 0.03 to 0.556 ± 0.03 (F6, F8), as demonstrated in Figure 2b. These results confirmed that the larger the particle size, the greater the PDI, and vice versa (Kaur et al., 2016a). Increasing PDI values upon increasing SAA% might be related to increasing the viscosity of the aqueous phase, which affected the emulsification efficiency during SLN preparation. As a result, particles of varying sizes were formed that contributed to a higher PDI (Hassan et al., 2020).

Increasing the homogenization speed from 10000 to 15000 rpm; resulted in an increase in PDI from 0.358 ± 0.03 to 0.528 ± 0.02 (F1, F2), 0.716 ± 0.07 to 1 ± 0.00 (F3, F4), and 0.191 ± 0.03 to 0.556 ± 0.03 (F7, F8) as distinct in Figure 2c. Similar findings were obtained by Anarjan and coauthors, who found that the PDI of the nanodispersion systems was increased by increasing the speed of the homogenization process (Anarjan et al., 2015).

#### 3.1.3. Zeta potential

Zeta potential is the overall charge of the particles, which helps to assess the formulation stability during storage (Radwan et al., 2019). The obtained results showed a non-linear correlation between ZP values and the independent variables, as observed in the following polynomial equation:

Y2 = −18.077−0.393 X1+ 0.143 X2 − 0.292 X3− 1.083 X1*X2 −0.967 X1*X3− 0.482 X2*X3

All SLN formulations showed ZP values with negative charges, which indicated the stable nature of nanoparticles (Remya & Damodharan, 2018). The negative charge of SLNs might be attributed to the fatty acids released from GMS hydrolysis and the negative phospholipids from lecithin (Schuh et al., 2014; Emami et al., 2015).

As demonstrated in Table 2, Val-loaded SLNs showed ZP ranges between −15.36 ± 0.51 mV and −22.06 ± 0.67 mV for F4 and F8, respectively. This ascertained better stability and dispersion in the medium (Shah et al., 2017).

### 3.2. Entrapment efficiency % (Y3)

Regression analysis equation that interprets the effect of independent variables on EE% (Y3) is represented as following:

Y3 = 41.49 + 6.00 X1+2.65 X2+3.02 X3−4.74 X1*X2−6.53 X1*X3+0.43 X2*X3

**Figure 3.**
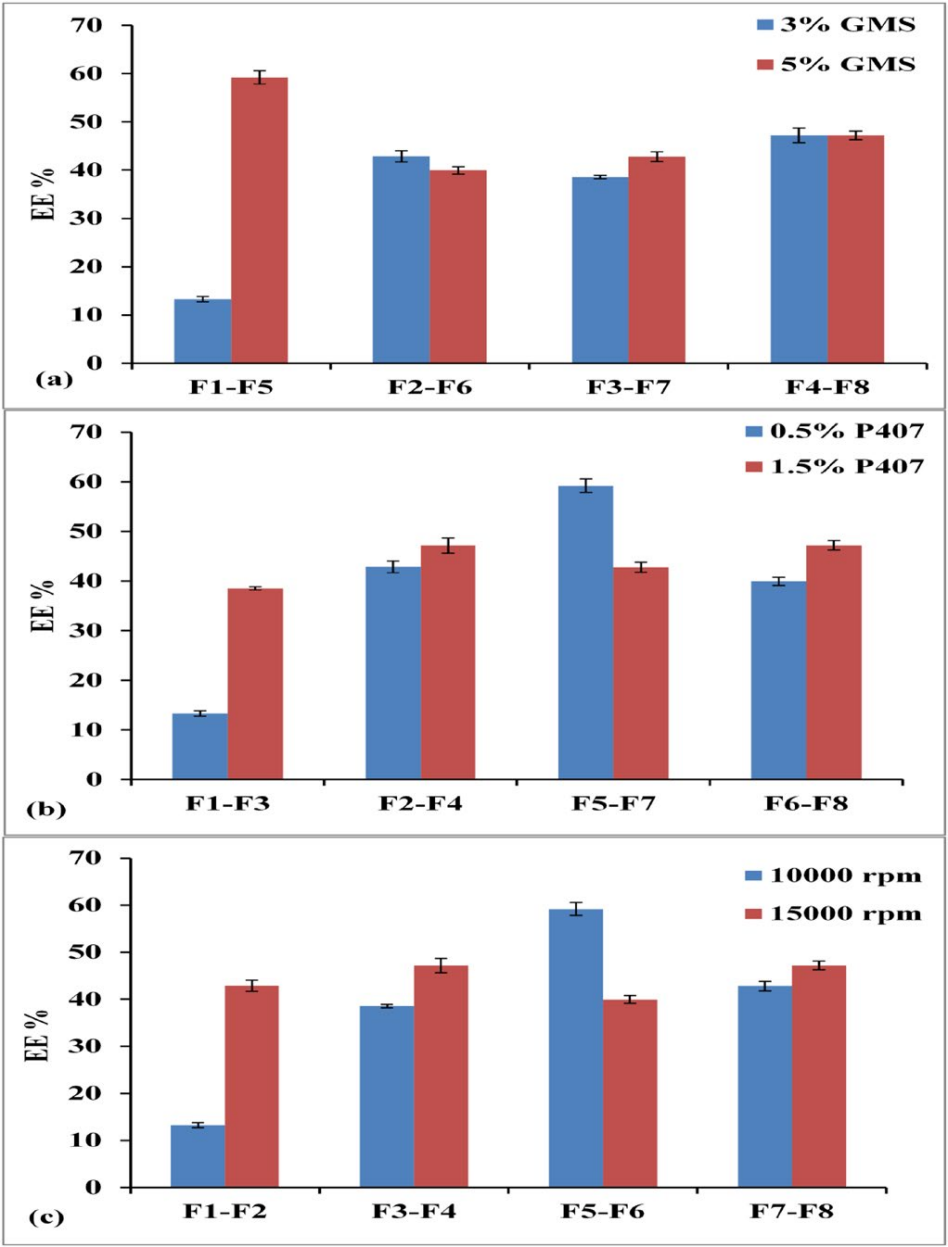
Effect of lipid % (a), SAA % (b) and homogenization speed (c) on EE%.

From the obtained results in Figure 3a, it was evident that an increase in the lipid ratio from 3 to 5% w/v led to increased EE% from 13.3 ± 0.521% to 59.2 ± 1.37% (F1, F5) and 38.56 ± 0.34% to 42.8 ± 0.99% (F3, F7) at constant SAA% and homogenization speed.

These results might be contributed to excessive drug accommodation within lipid core with high lipid concentration (Emami et al., 2015). Similar results were obtained by Emami et al., and Soma et al., who found that, increasing the lipid content, led to increasing the viscosity of the medium and faster solidification of nanoparticles, thus preventing drug diffusion into the external phase (Emami et al., 2015; Soma et al., 2017).

As observed in Figure 3b, there was a significant positive correlation between SAA concentration and EE%. Increasing P407% from 0.5 to 1.5% w/v led to a significant increase in EE% from 13.3 ± 0.521% to 38.56 ± 0.347% (F1, F3), 42.88 ± 1.17% to 47.2 ± 1.536% (F2, F4), and 39.95 ± 0.79% to 48 ± 0.92% (F6, F8). These results might be attributed to, increasing the surface coverage of SLNs by increasing the SAA% and thus preventing drug leaching from the lipid matrix and consequently increasing the EE% (Kushwaha et al., 2013; Maqsood et al., 2022(.

On the other hand, the combinatory effect caused by the simultaneous increase in the concentration of P407 in the aqueous phase decreased the EE% of Val from 59.2 ± 1.37% to 42.8 ± 0.99% (F5, F7) upon increasing SAA% from 0.5 to 1.5% w/v at (5% lipid and 10000 rpm). This was also observed by Da Silva et al. (2011) and Emami et al. (2015), who found that P407 might favor the solubilization of Val in the aqueous medium.

The obtained results in Figure 3c detected that, the increase in homogenization speed from 10000 to 15000 rpm at constant lipid and SAA %, led to a significant increase in EE% from 13.3 ± 0.52% to 42.88 ± 1.17% (F1, F2), 38.56 ± 0.34% to 47.2 ± 1.53% (F3, F4) and 42.8 ± 0.99% to 48 ± 0.92% (F7, F8). These results were also reported by Mai et al. (2018).

### 3.3. In-vitro release study

**Figure 4.**
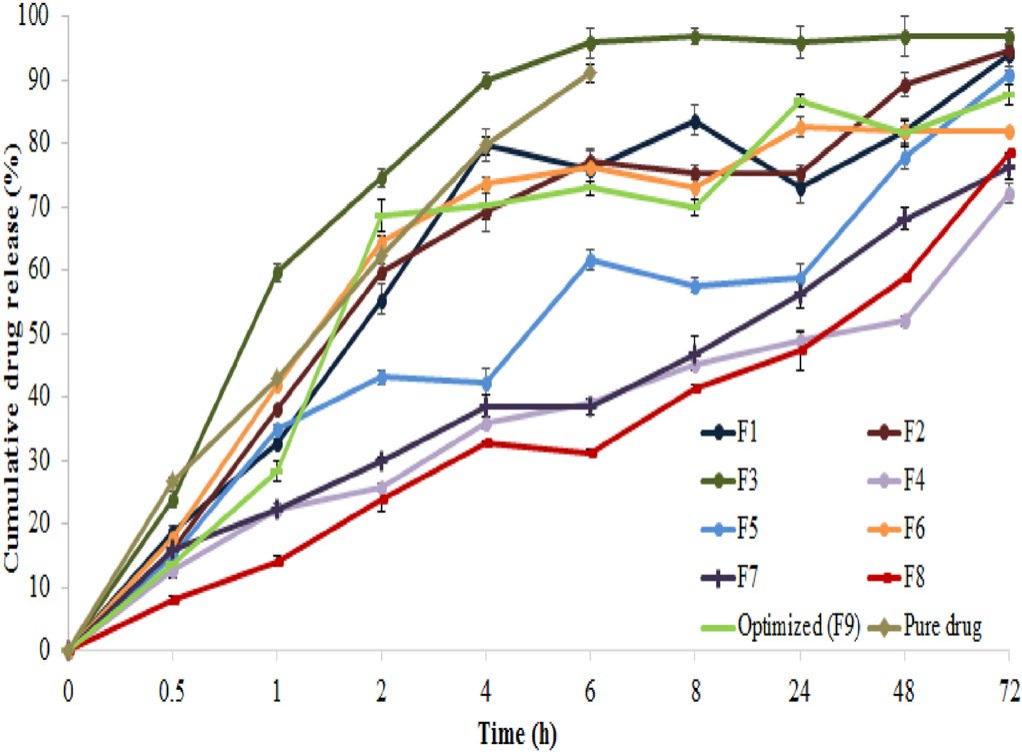
CDR % of all SLNs formulations compared to pure drug solution.

The *in-vitro* release of Val from pure drug solution and all SLN formulations was studied for 72 hours using the dialysis bag method in a BPS medium (pH 6.4). Pure Val showed a rapid release of about 90% in the first six hours, as observed in Figure 4. The effect of the independent factors on CDR% is illustrated in the following polynomial equation:

Y4 = 85.621− 3.726 X1 − 4.661 X2 − 3.831 X3 − 0.231 X1*X2 − 2.191 X1*X3 + 1.834 X2*X3

The release pattern of most SLN formulations was noticed to be biphasic, with an initial burst release within the first four hours, this might be attributed to the drug on the surface of SLNs and its solubility in the selected medium (Kaur et al., 2016b; Rana et al., 2020), followed by sustained release over 72 hours (Yassin et al., 2010; Remya & Damodharan, 2018).

The sustained release might be due to increasing the diffusional path length and hindering effects attained by the surrounding lipid core (Alajami et al., 2022).

There was a negative correlation between X1, X2, and X3 on CDR% as indicated by Pearson correlation coefficients (-0.418, −0.523 and −0.430), respectively.

For most formulations, increasing the lipid concentration, maintaining the drug release up to 72 hours by restricting the entry of medium and inhibited fast immobilization of Val from the lipid core and thus, extended the release % (Parvez et al., 2020). In another explanation, the diffusion distance from the lipid matrix decreased with the increase in lipid concentration (El-Say & Hosny, 2018; Bhattacharyya & Reddy, 2019).

### 3.4. Kinetic studies

As seen in Table 3, the Higuchi model had a higher R^2^ value for *in-vitro* drug release of all SLN formulations than the Hixson-Crowell model. In addition, R^2^ value for the first order model was higher than that of zero order. These findings established that the drug release from all formulations followed the diffusion mechanism (Ibrahim et al., 2021). The n value of the Korsmeyer-Peppas model provided additional confirmation about the type of diffusion (Bibi et al., 2022). As detected in Table 3, n-value was found to be less than 0.5, indicating that the drug release mechanism was quasi-Fickian diffusion (Basak et al., 2008; Olejnik et al., 2017).

**Table 3.** Kinetic studies of drug release from SLNs formulations and the optimized formula (F9).

Formulation code.	R2					n-value of Korsmeyer–Peppas model
	Zero	First	Higuchi	Korsmeyer	Hixon	
F1	−1.2561	0.8465	0.0219		−0.1506	
F2	−1.1221	0.8453	0.1484	0.8700	−0.0276	0.183
F3	−2.0670	0.9831	−0.4425	0.8106	−0.6813	0.129
F4	−0.2790	0.1229	0.6192	0.9456	−0.0204	0.251
F5	−0.4761	0.5519	0.5154	0.9307	0.2395	0.231
F6	−1.7367	0.7595	−0.2277	0.8248	−0.4008	0.147
F7	−0.1446	0.4398	0.7205	0.9888	0.2834	0.269
F8	0.3359	0.6126	0.8762	0.9654	0.5254	0.340
Optimized SLN (F9)	−1.1569	0.8045	0.0837	0.8049	−0.0298	0.179

### 3.5. Stability study of SLNs formulations

Color change, particle aggregation, or phase separation were not seen in stored SLN formulations. According to the findings in Table 4, the particle size of certain formulations decreased significantly after being stored at refrigerator temperature. The decrease in particle size was correlated with the increase in the ZP as observed in F1, F3, and F7 respectively. Decreasing the particle diameter on storage at refrigerator temperature might be attributed to the microviscosity phenomenon, which is a property of the surfactant that prevents particle agglomeration and is a temperature-dependent factor that increases at refrigerator temperature (Shah et al., 2014; Makoni et al., 2019).

**Table 4.** Effect of storage at 4ºC on PS, PDI and ZP of SLNs formulations.

Code	Particle size (nm)		PDI		Zeta potential (mV)	
	Fresh formulations	Stored formulations	Fresh formulations	Stored formulations	Fresh formulations	Stored formulations
F1	99.05 ± 4.62	89.35 ± 1.32*	0.358 ± 0.034	0.41 ± 0.03#	−19.16 ± 0.37	−17.7 ± 0.26*
F2	138.33 ± 1.89	133.23 ± 7.97#	0.528 ± 0.02	0.418 ± 0.01*	−18.66 ± 0.23	−17.60 ± 0.51#
F3	1925.60 ± 5.07	1644.40 ± 34.46*	0.716 ± 0.078	0.56 ± 0.03#	−17.56 ± 0.28	−14.46 ± 0.05*
F4	639.46 ± 39.71	713.83 ± 46.42#	1 ± 0.00	0.55 ± 0.03*	−15.36 ± 0.51	−14.93 ± 0.92#
F5	98.28 ± 5.63	150.67 ± 7.48*	0.259 ± 0.086	0.59 ± 0.01*	−17.66 ± 0.21	−15.63 ± 0.251*
F6	101.89 ± 2.84	123.46 ± 13.43#	0.195 ± 0.03	0.35 ± 0.06*	−17.4 ± 0.36	−16.16 ± 0.35#
F7	394.10 ± 18.37	336.80 ± 12.60	0.191 ± 0.036	0.58 ± 0.09*	−16.76 ± 0.81	−14.66 ± 0.37#
F8	213.63 ± 0.50	255.63 ± 39.70#	0.556 ± 0.036	0.316 ± 0.01*	−22.06 ± 0.66	−19.20 ± 0.20*

All values are represented as mean ± SD.

# non-significant; *significant.

### 3.6. Design optimization

As obvious in Table 5, the particle size of the optimized formulation (F9) without Rh-B coupling was higher than predicted (215.76 ± 7.47 nm to150 nm, respectively), but this value is still acceptable for brain targeting of SLNs, as demonstrated by Neves and coauthors, who reported that the particle size of most successfully used nanoparticles for drug delivery across the BBB ranged from 150-300 nm (Neves et al., 2015). However, particle size of (F9) which was loaded with Rh-B and administered in animals for *in-vivo* experiments was 166 ± 5.33 nm. Other measurements (PDI, ZP, and CDR%) were in good harmony with the predicted values as detectable in Table 5, which clarifies that the experimental design closely predicted the relationship between the dependent and independent variables and successfully assisted in setting up a model for optimizing Val-loaded SLNs (Gupta et al., 2016).

**Table 5.** Predicted and observed response values for optimized formulation (F9).

Response	Predicted value	Observed value
Y1: particle size (nm)	150	215.76 ± 7.47
Y2: zeta potential (mV)	−20	−15.26 ± 0.58
Y3: EE (%)	60	59.45 ± 0.88
Y4: CDR (%)	90	87.59 ± 1.67

### 3.7. Fourier transform infrared (FTIR) spectroscopy

**Figure 5.**
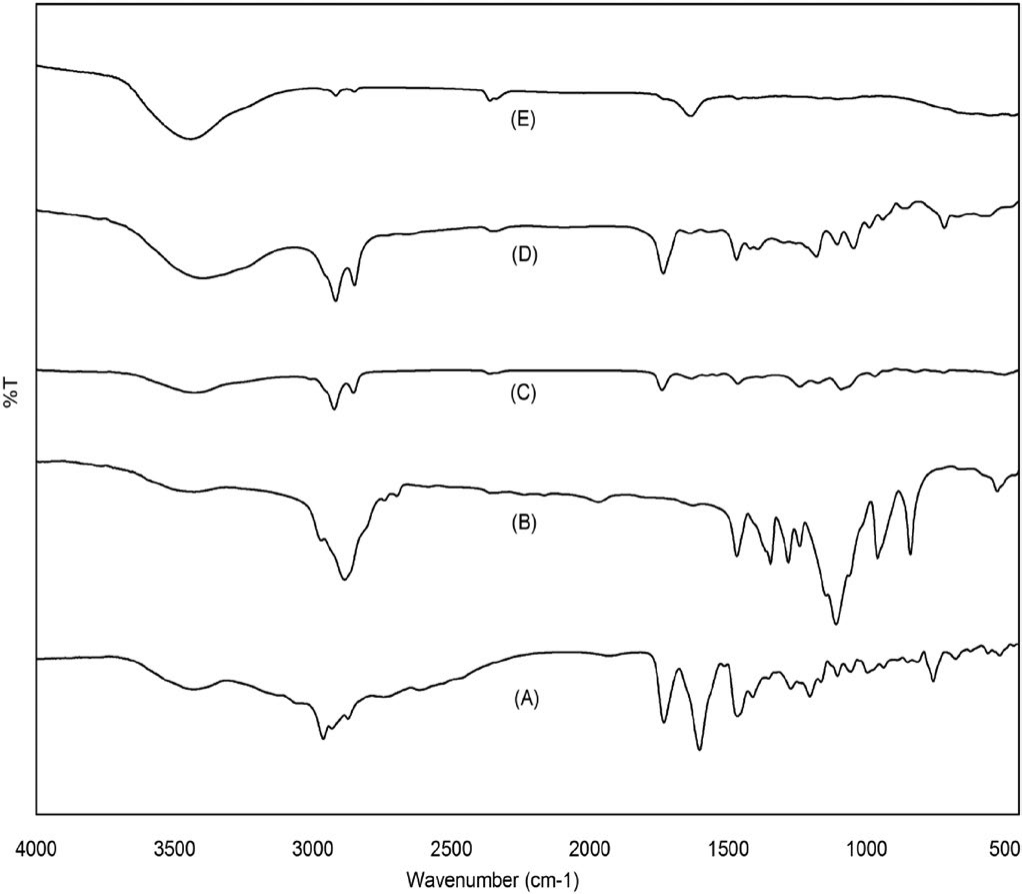
FTIR spectra of (a) Pure drug; (b) P407; (c) egg lecithin; (d) GMS; (e) optimized Val-loaded SLN (F9).

FTIR was performed to investigate the possible type of interaction between pure Val and optimized formula components by elucidating the reduction, shifting, or disappearance of absorption bands of the studied samples. Figure 5 compares the FTIR spectra of the pure Val, the optimized formula, and its raw materials.

In agreement with Chandana et al., Val showed characteristic peaks at 2963 cm^−1^ and 2932 cm^−1^ (C-H stretching, alkane), 1732 cm^−1^ (acidic C = O stretching), 1603 (ketonic C = O stretching), and 3430 cm^−1^ (carboxylic group, -COOH) (Chandana et al., 2021).

The spectrum of GMS showed absorption bands at 3398 cm^−1^ for O-H stretching from the unesterified hydroxyl group of the glyceryl moiety, 1734 cm^−1^ (C = O, stretching), and 2918 cm^−1^ for C-H stretch in CH2 groups in the acyl chain of the fatty acid (Patel et al., 2014).

In the case of the optimized formulation (F9), the characteristic peaks unique to the drug were not observed, indicating drug encapsulation into the nanocarrier (Ghorab et al., 2015; Omwoyo & Moloto, 2019). As well, there was a slight increase in the (O-H) band of GMS, which might be due to the formation of a hydrogen bond between Val and GMS.

### 3.8. Differential scanning calorimetry

**Figure 6.**
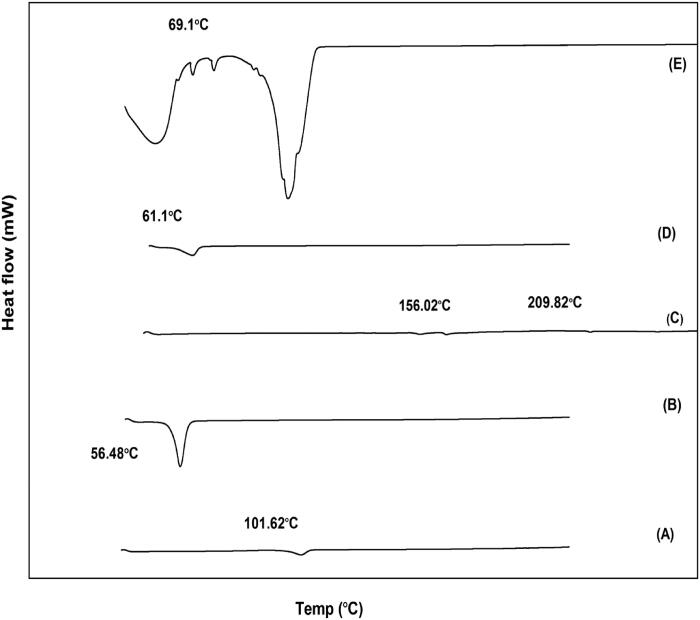
DSC thermogram of (a) Pure drug; (b) P407; (c) egg lecithin; (d) GMS; (e) optimized Val-loaded SLN (F9).

DSC patterns of Val, P407, egg lecithin, GMS, and optimized SLN (F9) were shown in Figure 6. It was clear that the melting peaks of bulk GMS and pure Val were at 61.1 °C and 101.62 °C, respectively. The sharp peak of Val crystals (101.62 °C) was absent in the thermogram of Val-loaded SLN (F9), which confirmed drug solubilization in the lipid matrix. Moreover, the endothermic peak of GMS in F9 was broadened and shifted to 69.1 °C compared to bulk GMS (61.1 °C), which indicated SLN formation (Sharma et al., 2021). The same manner was seen by Song and coworkers, who attributed this shift to the small particle size effect (nanometer range), their high specific surface area, and the presence of surfactant (Song et al., 2016).

### 3.9. X-ray diffraction study

**Figure 7.**
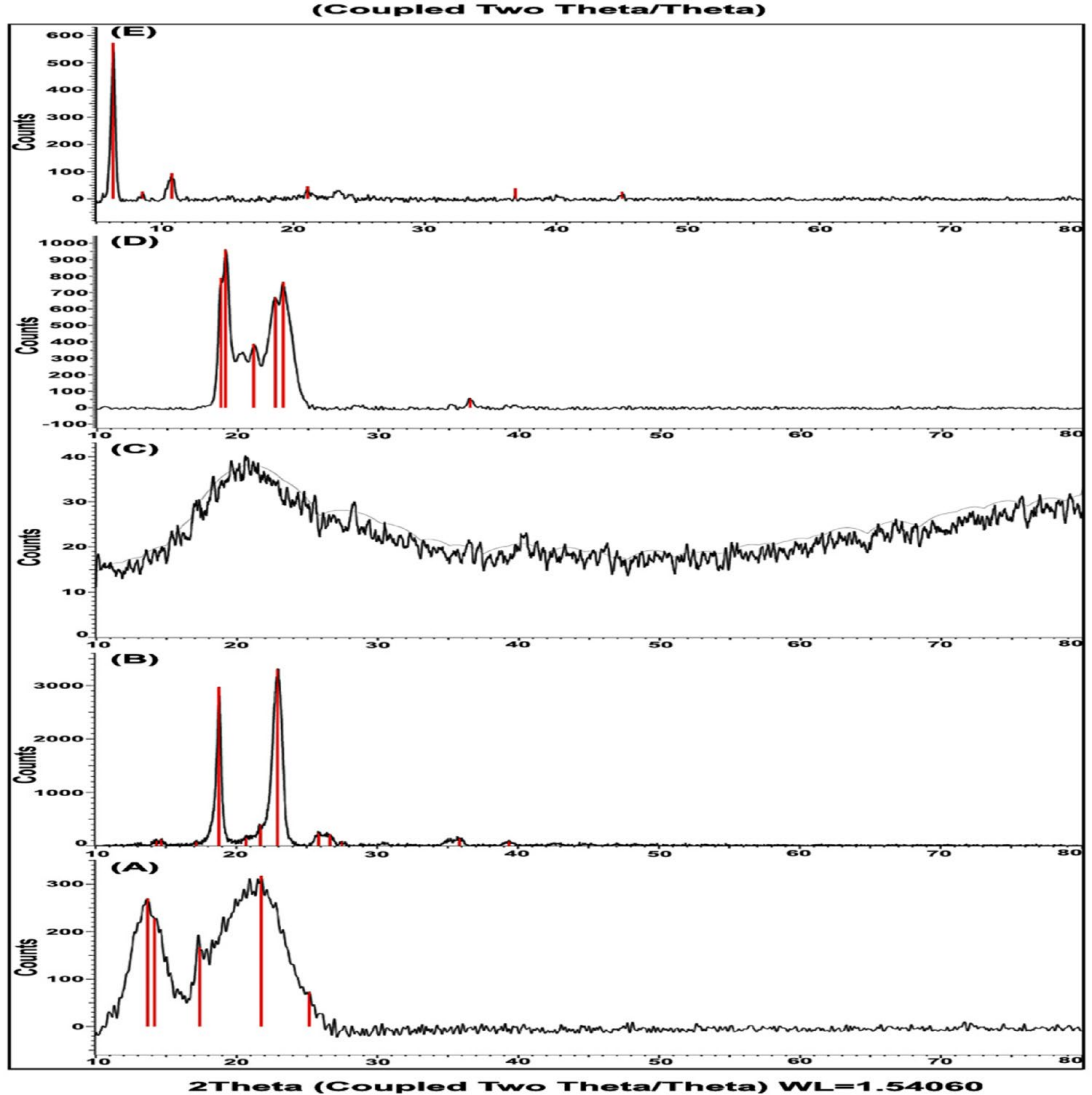
XRD spectra of (a) Pure drug; (b) P407; (c) egg lecithin; (d) GMS; (e) optimized Val-loaded SLN (F9).

The XRD spectra of pure Val, optimized SLN (F9), and its components are represented in Figure 7. The diffraction spectra of pure Val showed characteristic peaks at a diffraction angle of 2θ degrees of 13.696, 14.194, 17.393, 21.775, and 25.182. These results were in great agreement with Sharma & Jain (2010), Zaini et al. (2017), and Abbaspour et al. (2021). Glyceryl monostearate is high crystalline in nature with characteristic peaks at 2θ of 18.823, 19.137, 21.146, 22.676, 23.23 and 36.51 with the highest intensity peak at 19.137 representing the *β*-crystal form (Su et al., 2016).

The intensity of crystalline peaks of Val was reduced in the SLN formulation, which provided additional support that the drug was encapsulated within the carrier system (Parmar et al., 2011). The intensity of lipid peaks was also decreased in the SLN formulation which confirmed the decreased crystallinity of lipid in the SLN formulation (Parmar et al., 2011; Kushwaha et al., 2013; Rohit & Pal, 2013; Gupta et al., 2016; Behbahani et al., 2017).

### 3.10. Transmission electron microscope (TEM)

**Figure 8.**
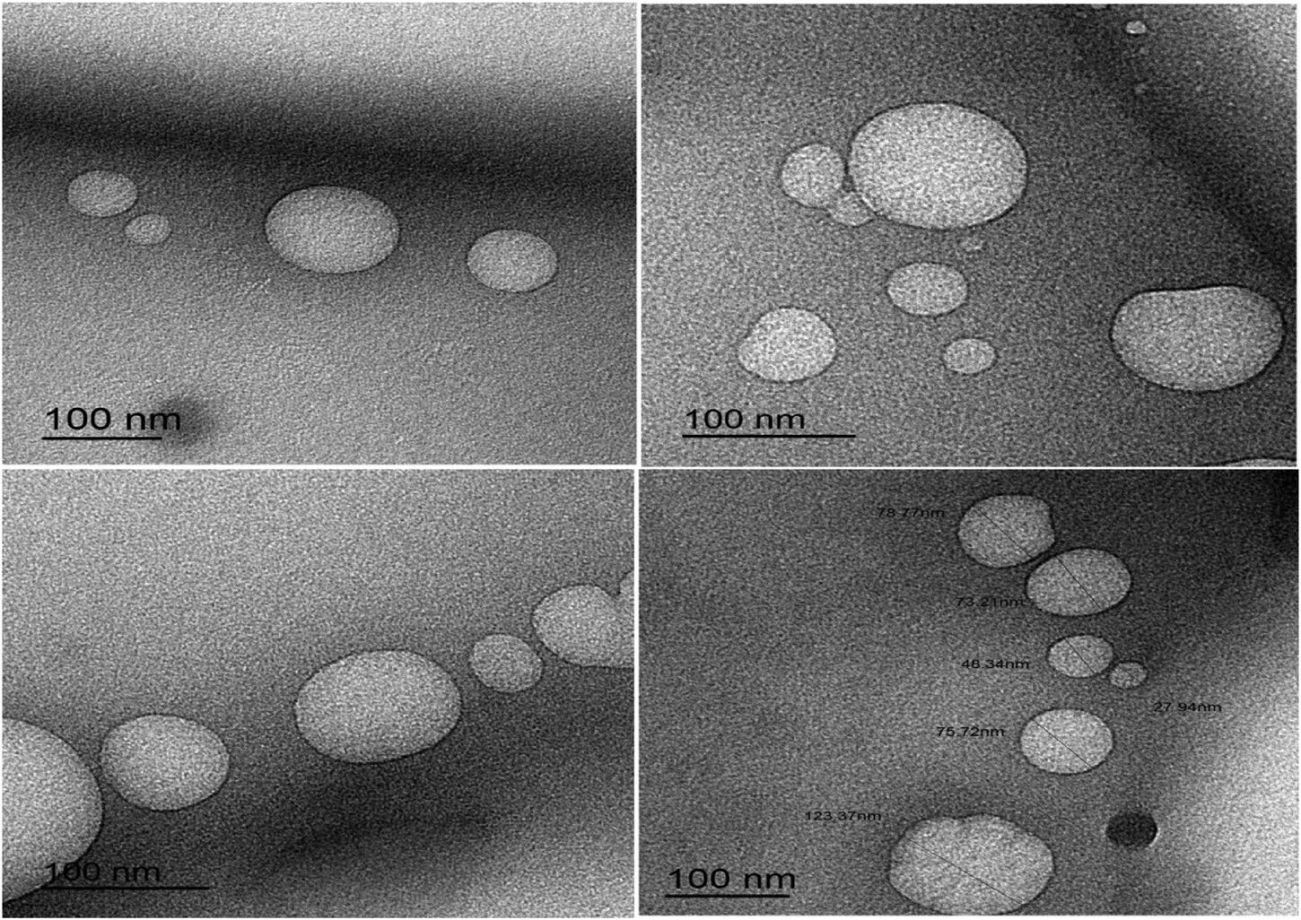
TEM images of optimized SLN formulation (F9) loaded with rhodamine B.

**Figure 9.**
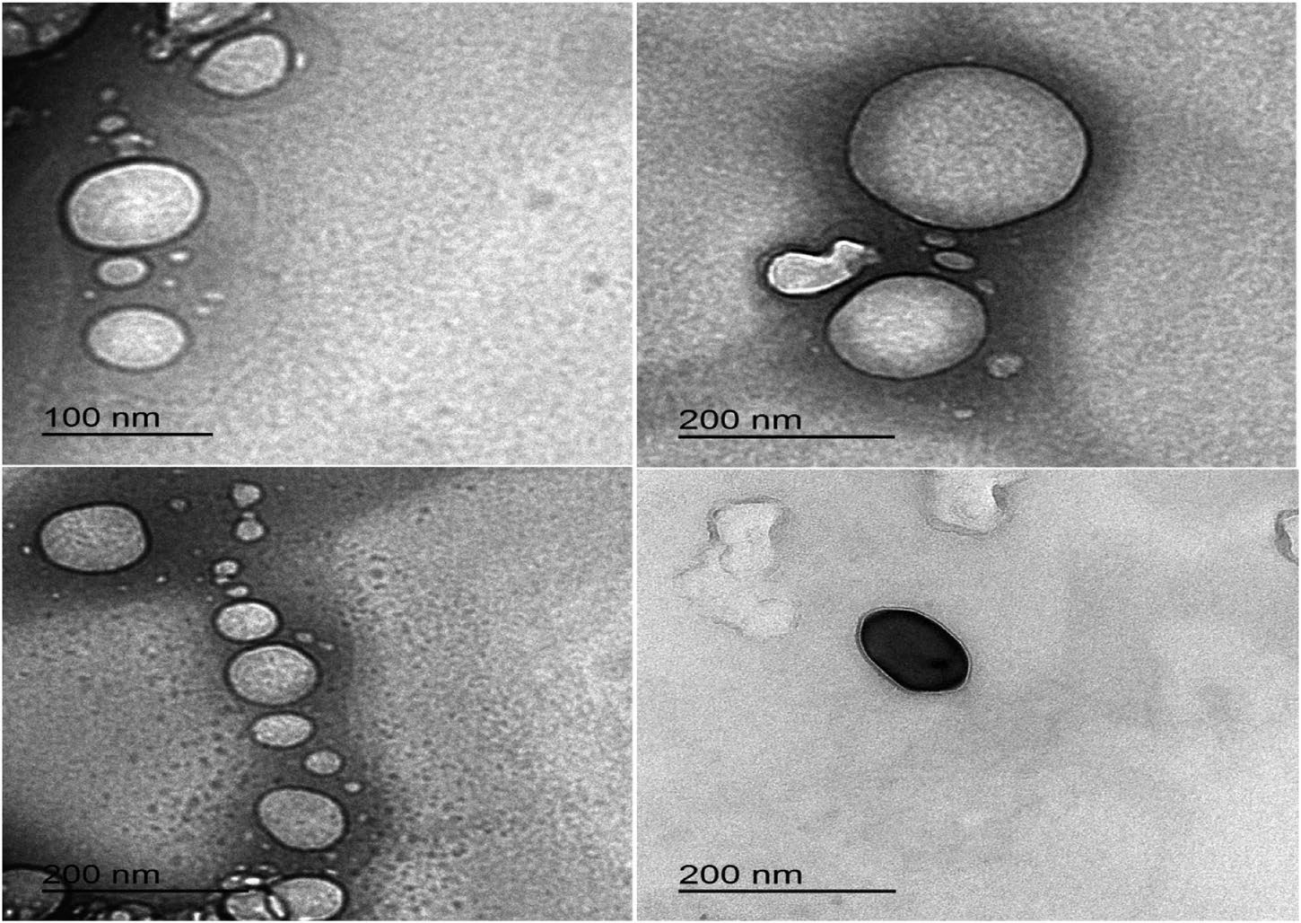
TEM images of optimized SLN formulation (F9) without rhodamine B.

The shapes of the optimal Val-loaded SLN formulation (F9) with and without Rh-B coupling are shown in Figures 8 and 9. The particles examined were spherical and homogenous in shape, with a coating encapsulating the nanoparticles. TEM images showed slightly smaller particle sizes of nanoparticles compared to those measured with a Zetasizer instrument based on dynamic light scattering (Rubab et al., 2021).

### 3.11. Fluorescence imaging and photon quantification

The fluorescent formulations were administered intranasally in mice, and fluorescence was investigated in the brain and lung to verify whether the drug was successfully distributed to the brain or not. The fluorescence intensity-per-area was identified by a color ranging from dark blue (low accumulation) to red (maximum accumulation) (El-Mezayen et al., 2018). Measurements were done by tracing a region of interest (ROI) on the fluorescent images, utilizing (PhotoAcquisition M3Vision analysis software) for photon quantification. These measurements were performed for lung and brain for all *in-vivo* tested formulations (Mannucci et al., 2020).

**Figure 10.**
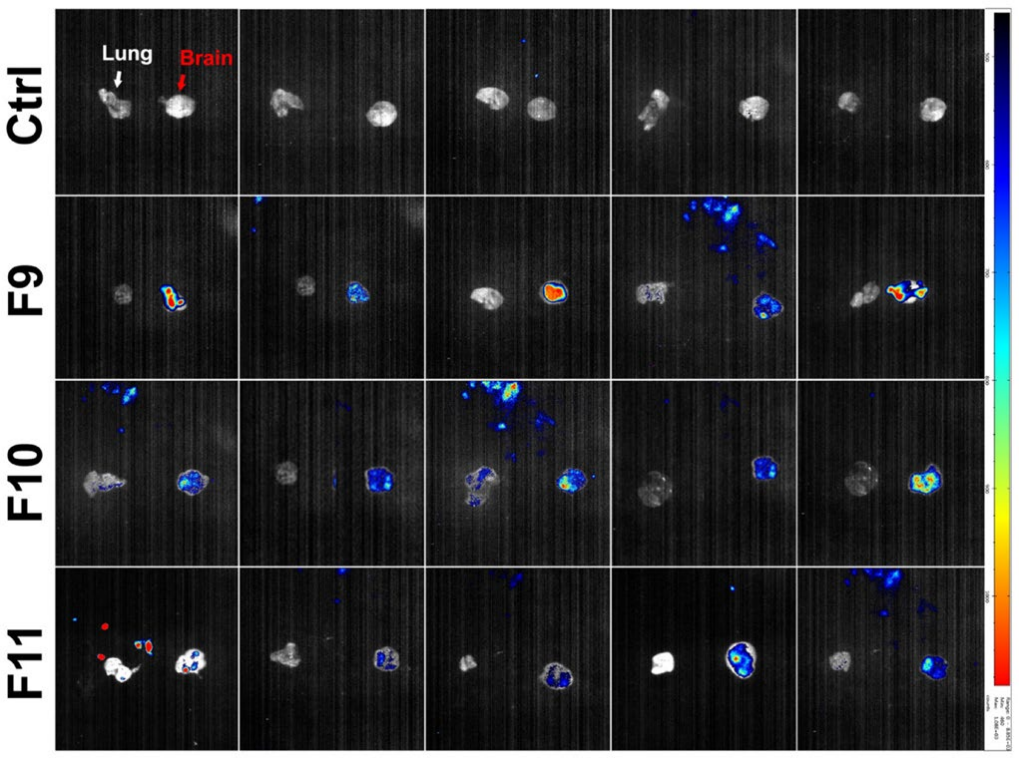
Fluorescent images acquired by Photon-imager of dissected brain and lung of: **F9**: SLN loaded with Val F10: Blank SLN; **F11:** Pure Val solution; **N.B.** organ in the right side of the image referred to the brain and organ in the left side referred to the lung.

The obtained results in Figure 10 showed that mice received the optimized formulation (F9) showed an apparent red fluorescence signal at the brain site, reflecting higher drug accumulation in the brain and successful delivery of Val loaded nanoparticle formulation across the BBB, as compared to the mice received the pure drug solution (F11), which showed a blue fluorescence at the brain site indicated the poor permeability of the pure drug across the BBB. These results were additionally confirmed by quantifying the fluorescence intensity in the brain and the lung.

**Figure 11.**
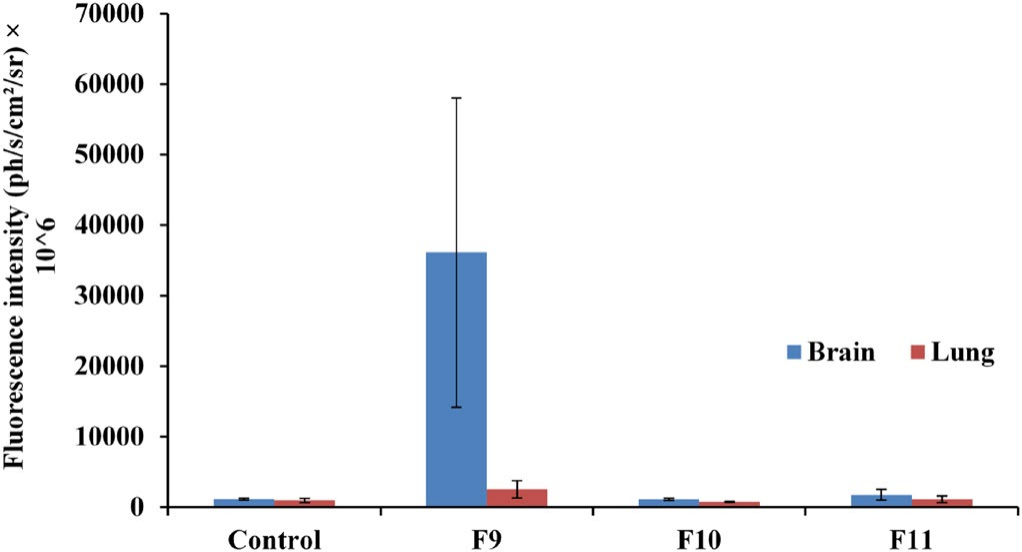
Fluorescence intensity in brain and lung of: **F9:** SLN loaded with Val **F10:** Blank SLN **F11:** Pure Val solution.

The obtained results in Figure 11 depict that the optimized SLN formulation (F9) showed significantly higher fluorescence intensity in the brain as compared to the pure Val solution (F11). Furthermore, there was a non-significant difference (*P* > 0.05) in florescence intensity between F11 and the control group which confirmed poor delivery of pure Val to brain. The organ distribution study revealed a higher accumulation of optimized formula in the brain as compared to free drug solution, confirming the successful delivery of Val-loaded SLN formulation to the brain.

## 4. Conclusion

The results of various experiments led to the conclusion that the optimal SLN formula (F9), as suggested by full factorial design (2^3^) was at a lipid concentration of 4.9379% w/v, 0.6507% w/v P407, and 10,000 rpm, demonstrated acceptable particle size, EE%, and sustained drug release over three days, which could be beneficial in decreasing dose frequency and increasing patient compliance. In-vivo photon imaging and fluorescence intensity quantification in dissected brain and lung of all animal groups indicated that the optimized Val-loaded SLN successfully delivered Val to the brain. Finally, Val-loaded SLNs could be a promising strategy for mitigating the negative effects of stroke with high efficacy and low side effects.
